# Comments on a recent case-control study of malignant mesothelioma of the pericardium and the tunica vaginalis testis

**DOI:** 10.5271/sjweh.3909

**Published:** 2020-12-16

**Authors:** Gabor Mezei, Ellen T Chang, Fionna S Mowat, Suresh H Moolgavkar

As the first case–control study of malignant mesothelioma of the pericardium and the tunica vaginalis testis (mTVT), the paper by Marinaccio et al (1) is potentially an important epidemiologic contribution. A careful review of the paper, however, raises a number of methodological issues.

Any case–control study can be viewed as being nested within a conceptual cohort, with controls being sampled from the at-risk cohort as cases arise over time. This view of case–control studies leads to the concept of incidence-density sampling of controls (eg, 2, 3). For Marinaccio et al (1) this would mean that, as cases were registered over the study period, each would be matched to an individual control or set of controls of the same gender, age, and region of the country (since asbestos exposure varies by time and region [4]). For example, if a case were 50 years old in 1995, then any matched control should be close to age 50 in 1995 and of the same gender and from the same region as the case. Matching for age in this fashion automatically results in matching for year of birth, which is essential in this context because birth-cohort effects are determinants of asbestos exposure and mesothelioma incidence (eg, 5–8). If Marinaccio et al (1) used this scheme for age-matching, one would expect to see similar distributions of cases (table 1) and controls (table S3 in the supplemental material) by period of birth. Among males, however, the distributions of mesothelioma cases (whether pericardial or mTVT) and controls by period of birth are clearly different (P<0.001). Among females, the distributions of cases of pericardial mesothelioma and controls by birth year are less dissimilar (P≈0.05). Thus, the female cases of pericardial mesothelioma are better matched to controls on year of birth than are male cases of either mTVT or pericardial mesothelioma. We note also that the distributions of male and female *controls* by year of birth are distinctly different (P<0.002), whereas the birth-year distributions of *cases* of mesothelioma by site and gender are not (P≈0.8).

In the Marinaccio et al (1) sensitivity analysis restricted to subjects born before 1950, the distributions of cases and controls by period of birth remain significantly different. Therefore, based on the reported evidence, cases and controls were not matched on birth cohort, thereby possibly biasing the results. Similarly, bias may result from the lack of matching on geographic region; while cases were registered from across Italy, controls were selected from only six regions. Although a sensitivity analysis restricted cases and controls to those from only the six regions, a comparison of tables S1 and S3 indicates that the regional distribution of controls is different from that of person-time observed; that is, the controls do not appear to be representative of the underlying population at risk by region.

The second major issue of concern has to do with ascertainment of asbestos exposure. Information on exposure for the cases was presumably obtained at the time of registration. The two sets of controls, obtained from previously unpublished case-control studies, were interviewed during 2014–2015 and 2014–2016; that is, many years after the exposure for most cases was ascertained (1993–2015). Few other details of the control groups are provided, except that participation by one set of controls was <50%, raising additional concerns about selection bias. For details on the second set of controls, Marinaccio et al (1) reference a paper by Brandi et al (9). On review of that paper, however, we found no description of the control group, only references to three earlier papers. Marinaccio et al (1) present analyses only with both sets of controls combined; to evaluate potential sources of bias from the use of different sets of controls, they should also report results using each set of controls separately.

The authors also did not detail their methods of exposure classification. For example, what does probable or possible exposure mean? The authors should at least present separate analyses of definite occupational exposure. Eighty cases of mTVT were registered, but only 68 were included in the analyses. Information on the 12 omitted cases (eg, age, year of birth, and region) would be helpful. Marinaccio et al (1) did not provide clear information on what occupations and/or industries they considered as exposed to asbestos. In an earlier study, Marinaccio et al (10) remarked on the absence of pericardial mesothelioma and mTVT in industries with the highest exposures to asbestos, saying, “[t]he absence of exposures in the shipbuilding, railway and asbestos-cement industries … for all the 67 pericardial and testicular cases is noteworthy but not easy to interpret.” By contrast, Marinaccio et al (1) stated, “[t]he economic sectors more frequently associated with asbestos exposure were construction, steel mills, metal-working industry, textile industry and agriculture.” The possibility of exposure in the “agriculture economic sector” was not mentioned in Marinaccio et al (10) and appears not to have been considered in previous epidemiologic studies in Italy. In general, epidemiologic studies indicate that farmers and agricultural workers are not at increased risk of developing mesothelioma (eg, 11–17).

The fact that few, if any, cases of mTVT and pericardial mesothelioma occurred in industries traditionally associated with high asbestos exposure raises the possibility that the results of Marinaccio et al (1) are attributable to deficiencies in study design, very possibly bias in the selection of controls, and deficiencies in exposure assessment and classification as described above, leading to a spurious association of occupational exposure with mTVT and male pericardial mesothelioma.

## Conflict of interest

This research has received no outside funding. All authors are employees of Exponent, Inc., an international scientific and engineering consulting company. All authors have worked as both consulting and testifying experts in litigation matters related to asbestos exposure and asbestos-related disease.
